# Dietary Fat and Carbohydrate Exposure During a Group-Based Nutritional Psychoeducational Program in Anorexia Nervosa

**DOI:** 10.3390/nu18060902

**Published:** 2026-03-12

**Authors:** Paolo Meneguzzo, Alessandra Zattarin, Arianna Carpin, Anna Svaizer, Beatrice Varotto, Zaira Salvador, Anna Marchetto, Angela Veronese, Angela Favaro

**Affiliations:** 1 Department of Neuroscience, University of Padova, 35128 Padova, Italy; 2 Padova Neuroscience Center, University of Padova, 35128 Padova, Italy; 3 Eating Disorders Center, Azienda Ospedale-Università of Padova, 35128 Padova, Italy; 4 Clinical Nutrition Unit, Azienda Ospedale-Università of Padova, 35128 Padova, Italy; 5 Department of Medicine-DIMED, University of Padova, 35128 Padova, Italy

**Keywords:** anorexia nervosa, nutritional psychoeducation, eating behavior, food frequency questionnaire, dietary rules, nutritional knowledge, day hospital

## Abstract

**Background:** Nutritional psychoeducation is a core component of multidisciplinary treatment for anorexia nervosa, yet evidence on its association with changes in eating behavior beyond weight outcomes remains limited. **Methods:** This pre–post observational study included 45 patients with anorexia nervosa attending a Day Hospital program who participated in a structured, group-based nutritional psychoeducational intervention as part of standard multidisciplinary care. Nutritional knowledge, dietary rules, eating behaviors, food group exposure assessed by a Food Frequency Questionnaire (FFQ), and body mass index (BMI) were evaluated before and after the intervention. Pre–post changes were examined using paired statistical tests with Holm correction. Associations between changes in cognitive–nutritional variables and eating behavior were explored using correlations and multiple linear regression models. **Results:** Significant pre–post improvements were observed in nutritional knowledge and reductions in rigid dietary rules. Eating behavior showed specific changes, with increased exposure to carbohydrate- and fat-containing foods, as well as improved meal adequacy. BMI increased during the observation period, consistent with expected outcomes of Day Hospital treatment. Changes in nutrient-related knowledge were positively associated with changes in dietary fat exposure, independent of baseline BMI and changes in dietary rules, whereas no comparable association was observed for carbohydrate exposure. **Conclusions:** In this Day Hospital sample, participation in a group-based nutritional psychoeducational program within a multidisciplinary treatment context was associated with specific changes in eating behavior alongside cognitive–nutritional changes and weight gain. The observed association between nutrient-related knowledge and dietary fat exposure may suggest the relevance of assessing food-specific behaviors and cognitive–nutritional processes as complementary outcomes during treatment for anorexia nervosa.

## 1. Introduction

Anorexia nervosa (AN) is a severe psychiatric disorder characterized by persistent concerns about body weight and shape, restrictive eating behaviors, and significantly low body weight, with substantial medical and psychological consequences [1]. Lifetime prevalence estimates indicate rates of approximately 4% among females and 0.3% among males, with recent epidemiological evidence suggesting an increasing incidence among children and adolescents [2,3].

According to the transdiagnostic cognitive–behavioral model of eating disorders, rigid dietary rules, caloric restriction, and food avoidance play a central role in maintaining anorexia nervosa by reinforcing dysfunctional beliefs about control, self-worth, and weight regulation [4]. These processes are further sustained by additional maintaining factors, including perfectionism, low self-esteem, mood intolerance, and interpersonal difficulties [5,6]. Within this framework, eating behavior is not only a behavioral manifestation of the disorder but also a key target for therapeutic change.

International clinical guidelines consistently recommend a multidimensional, interdisciplinary treatment approach for anorexia nervosa, in which nutritional rehabilitation represents a core component across levels of care [7,8]. Importantly, nutritional rehabilitation within cognitive–behavioral approaches is not limited to weight restoration but also aims to modify dysfunctional beliefs about food, challenge rigid dietary rules, and reduce avoidance of specific food groups [9,10,11]. In clinical practice, particularly within Day Hospital settings, treatment is typically delivered by a multidisciplinary team including psychiatrists, psychologists, dietitians, and nursing staff. Interventions commonly integrate medical monitoring, nutritional rehabilitation, individual and group psychotherapy, and skills-based interventions targeting emotional regulation and interpersonal functioning. Within this coordinated framework, nutritional psychoeducation represents one component aimed at addressing maladaptive cognitions and behaviors related to food.

Psychoeducation constitutes an integral element of cognitive–behavioral treatments for eating disorders, including Cognitive–Behavioral Therapy (CBT) [12]. It is defined as an educational intervention designed to enhance patients’ understanding of the disorder, its maintaining mechanisms, and the rationale underlying treatment strategies, thereby supporting engagement in behavioral change. Within CBT, nutritional psychoeducation is closely integrated with behavioral and cognitive interventions and is intended to facilitate patients’ awareness of how restrictive eating patterns and dietary rules contribute to the persistence of the disorder [13,14].

Despite its widespread clinical use and strong theoretical rationale, empirical evidence specifically addressing the effects of structured nutritional psychoeducation on eating behavior and dietary patterns in patients with anorexia nervosa remains limited. Recent reviews have highlighted substantial heterogeneity in study designs and outcomes, with relatively few studies focusing on behavioral and nutritional changes beyond weight outcomes [15,16,17].

Against this background, the present study aims to evaluate the effects of a structured, group-based nutritional psychoeducational intervention delivered within a Day Hospital setting for patients with anorexia nervosa. Specifically, the study examines pre–post changes in nutritional knowledge, dietary rules, eating behaviors, and body mass index (BMI), with the objective of describing how changes in cognitive–nutritional domains are accompanied by changes in eating behavior during treatment.

## 2. Materials and Methods

### 2.1. Participants and Setting

The study included all patients diagnosed with anorexia nervosa who were admitted to the Eating Disorders Day Hospital at the Azienda Ospedale—Università di Padova between January 2022 and October 2024. Inclusion criteria were: (a) a diagnosis of anorexia nervosa according to DSM-5 criteria and (b) participation in at least four out of six scheduled nutritional psychoeducational sessions. All participants received standard multidisciplinary Day Hospital treatment alongside the psychoeducational intervention.

During the study period, 45 patients with anorexia nervosa participated in the psychoeducational program and met inclusion criteria. All 45 completed the baseline (T0) assessment, initiated the intervention, and completed the six-session program and the post-intervention (T1) assessment. In addition to the structured nutritional psychoeducational program, all patients received standard multidisciplinary Day Hospital treatment, including medical and psychiatric monitoring, individual psychotherapy, group psychotherapy sessions (including CREST-based emotional skills training [18]), dietetic counseling, and structured nursing support. These components were part of routine clinical care and were not manipulated for research purposes.

### 2.2. Nutritional Psychoeducational Program

During Day Hospital admission, all enrolled patients participated in a structured nutritional psychoeducation program consisting of six weekly group sessions, each lasting approximately one hour and conducted by a registered dietitian.

The sessions addressed the following topics:

- Session 1: Basal metabolism and determinants of energy requirements; physical and psychological consequences of underweight; cognitive dietary restriction and dichotomous food rules.
- Session 2: Carbohydrates and dietary fiber: sources, requirements, physiological functions, and consequences of inadequate intake.
- Session 3: Lipids: sources, requirements, functions, and the role of essential fats, with particular attention to body composition and amenorrhea.
- Session 4: Proteins: sources, requirements, functions, and consequences of excessive intake.
- Session 5: Vitamins and minerals: general characteristics and functions, with focused discussion on vitamin D, vitamin B12, calcium, sodium, potassium, and iron.
- Session 6: Water balance and the Harvard Healthy Eating Plate: hydration, fluid balance, and composition of a balanced meal.

Each session followed a consistent structure including: initial group discussion and reflection on previous content; interactive learning through questions, discussion, and shared beliefs; identification and discussion of common dietary myths relevant to the session topic; a brief knowledge quiz to reinforce key concepts; and homework assignments aimed at promoting gradual exposure to previously avoided foods and translating knowledge into everyday eating behavior.

### 2.3. Clinical Assessment

Body weight was measured and BMI calculated at Day Hospital admission, at least one week prior to the start of the psychoeducational program (T0), weekly during the intervention, and one week after completion of the six-session program (T1). Patients were admitted to the Day Hospital on a rolling basis rather than as a single cohort. Following admission, an initial phase of medical and nutritional stabilization occurred. The structured nutritional psychoeducational intervention began subsequently (T0), once patients were clinically stable and scheduled into an available group cycle. The interval between admission and T0 averaged 0.93 months (SD = 1.09; range 0–4 months), reflecting variability in clinical stabilization and group scheduling. Thus, BMI at admission and BMI at T0 represent distinct and individually variable clinical timepoints. T1 corresponds to the completion of the six-session psychoeducational intervention (approximately six weeks after T0).

### 2.4. Questionnaires

At T0 and T1, participants completed three anonymized self-report instruments.

Dietary Rules Inventory (DRI), a 28-item questionnaire assessing the presence and frequency of dietary rules over the previous 28 days [19]. Items are rated on a 5-point Likert scale (0 = never to 4 = always), yielding a total score and four subscales: what to eat, social eating, how much and when to eat, and caloric level.

Food Frequency Questionnaire (FFQ): a validated FFQ was used to assess weekly frequency of consumption of major food groups, including carbohydrate-containing foods, fat-containing foods, and protein sources, dairy products, fruits and vegetables, sweets, and fast food [20]. The FFQ assesses broad food categories commonly available in Mediterranean dietary contexts and was used to evaluate within-subject change rather than absolute intake estimation.

Dietary habits and nutritional knowledge were assessed using a self-report questionnaire specifically developed for the present study by the Day Hospital dietitians to capture changes targeted during the psychoeducational intervention. Items were generated to reflect the predefined content of the six psychoeducational sessions and commonly observed nutritional misconceptions in patients with anorexia nervosa in routine clinical practice. The preliminary item pool was reviewed by two senior clinicians and one registered dietitian to ensure clinical relevance and alignment with session objectives.

The questionnaire includes two sections.

(a) Dietary knowledge (37 dichotomous items) assessing nutritional myths, food composition, and topics addressed during the sessions. Items included true/false statements (e.g., “Carbohydrates should not be consumed in the evening”) and single- or multiple-choice questions regarding macronutrient functions, recommended percentage energy distribution (e.g., carbohydrate and lipid intake), and nutrient sources. Items were scored as correct (1) or incorrect/“don’t know” (0), with “don’t know” responses coded as incorrect to reflect absence of accurate knowledge. A total knowledge score was computed (range: 0–37). Internal consistency of the 37-item knowledge section in the present sample was acceptable (Cronbach’s α = 0.75). Given that the items were designed to cover distinct educational domains rather than to represent a single latent construct, subscale internal consistency indices were not emphasized.
(b) Dietary habits (17 items) assessing daily or weekly frequency of consumption of key food categories (e.g., starch-based foods, meat, fish, dairy, sweets), number of daily meals and snacks, typical meal composition (e.g., presence of first course, second course, fruit, vegetables), and contextual aspects of eating (e.g., shared meals, meal preparation responsibility). Frequency responses were converted into estimated times per week and aggregated into domain-specific indices (e.g., carbohydrate and fat exposure). For example, carbohydrate exposure was derived from reported daily frequency of starch-based foods (e.g., bread, pasta, rice), whereas fat exposure was indexed through frequency of consumption of lipid-dense foods and relevant meal components targeted during the intervention. Additional indicators captured clinically relevant patterns, including carbohydrate exposure and meal adequacy. Higher scores indicate greater exposure and more regular eating patterns. These indices represent aggregated behavioral frequency composites rather than reflective psychometric constructs; therefore, internal consistency metrics were not considered conceptually applicable.

The original Italian version is available from the authors upon reasonable request.

To clearly distinguish the two instruments, the FFQ provided a validated assessment of general weekly food frequency across broad food categories, whereas the study-specific habits questionnaire was designed to capture targeted behavioral changes aligned with the psychoeducational intervention (e.g., structured exposure to previously avoided macronutrient-containing foods and meal adequacy patterns). While both instruments assess aspects of eating behavior, they address partially distinct levels of measurement: the FFQ reflects standardized dietary frequency assessment, whereas the habit-derived indices were constructed to monitor clinically meaningful exposure processes within treatment.

### 2.5. Statistical Analysis

Descriptive statistics are reported as means and standard deviations or medians and interquartile ranges (IQR), as appropriate, for continuous variables and as frequencies and percentages for categorical variables. Normality of distributions and of pre–post difference scores was evaluated using visual inspection and the Shapiro–Wilk test. Pre–post changes in nutritional knowledge, dietary rules, eating behaviors, food frequency questionnaire (FFQ) domains, and body mass index (BMI) were examined using paired-sample *t* tests for normally distributed variables and Wilcoxon signed-rank tests for non-normally distributed variables. Effect sizes were calculated as Cohen’s dz for paired *t* tests and as r (|Z|/√N) for Wilcoxon tests. To control for multiple comparisons across primary outcomes, Holm’s correction was applied. To explore associations between changes in cognitive–nutritional domains and changes in eating behavior, change scores (Δ = post − pre) were computed for all relevant variables. Associations between change scores were examined using Spearman’s rank correlation coefficients. Multiple linear regression models were conducted to examine whether changes in nutrient-related knowledge and dietary rules independently predicted changes in fat exposure, adjusting for baseline BMI. Changes in nutrient-related knowledge and dietary rules were selected a priori as they represent the primary cognitive targets of the psychoeducational intervention and were hypothesized to be associated with behavioral change. Baseline BMI was included as a clinically relevant covariate. Given the modest sample size, the number of predictors was intentionally restricted to these theoretically driven variables to reduce the risk of overfitting and preserve model stability. All statistical tests were two-tailed, and the significance level was set at *p* < 0.05. Analyses were conducted using JASP (vers 0.95.4).

## 3. Results

### 3.1. Sample Characteristics

A total of 45 patients with a diagnosis of anorexia nervosa completed the nutritional psychoeducational program and both pre- and post-intervention assessments. Baseline characteristics are reported in Table 1. At admission, participants presented with low BMI, high levels of dietary rules, and restrictive eating patterns consistent with a Day Hospital population.

### 3.2. Changes in Eating Behavior and Food Exposure

**Figure 1:**
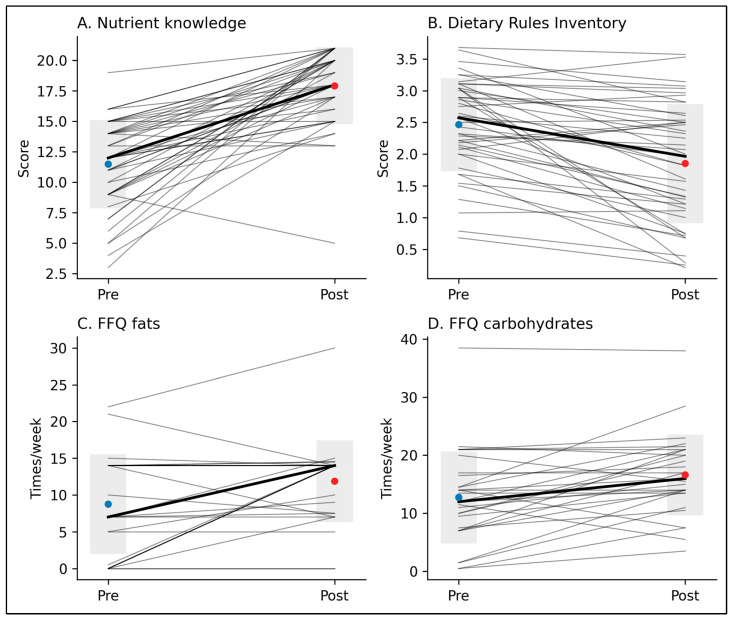
Individual pre–post changes in nutritional knowledge, dietary rules, and food group exposure. Panels display individual trajectories (thin gray lines) and group mean change (black line) from pre- to post-intervention. Blue dots indicate pre-intervention means and red dots post-intervention means; shaded areas represent ±1 standard deviation. (**A**) Nutrient-related knowledge score; (**B**) Dietary Rules Inventory total score; (**C**) weekly frequency of fat-containing foods assessed by the Food Frequency Questionnaire (FFQ); (**D**) weekly frequency of carbohydrate-containing foods assessed by the FFQ.

Pre–post analyses showed significant changes in eating behavior, particularly in food group exposure (Table 2). Participants significantly increased the weekly frequency of consumption of carbohydrate-containing foods (dz = 0.69, *p* = 0.003, Holm-corrected) and fat-containing foods (r = 0.46, *p* = 0.014). No significant changes were observed for other FFQ domains after correction for multiple testing. Consistent with FFQ findings, habit-based measures indicated a significant increase in carbohydrate exposure (r = 0.51, *p* < 0.001) and a significant improvement in meal adequacy (r = 0.48, *p* = 0.003), indicating more regular eating patterns. Individual trajectories for carbohydrate and fat exposure are shown in Figure 1, highlighting both overall increases and marked inter-individual variability.

### 3.3. Changes in Nutritional Knowledge and Dietary Rules

Nutritional knowledge increased significantly following the intervention. In particular, the nutrient-related knowledge subscale increased from 11.49 ± 3.63 at baseline to 17.91 ± 3.15 post-intervention (dz = 1.38, *p* < 0.001), and total nutritional knowledge scores showed a comparable increase (22.97 ± 5.98 to 32.24 ± 3.84, *p* < 0.001). Dietary rules, assessed using the Dietary Rules Inventory (DRI), decreased significantly from pre- to post-intervention (2.46 ± 0.73 to 1.85 ± 0.94; r = 0.83, *p* < 0.001), with reductions observed across all subdomains. BMI also increased significantly during the intervention period (16.17 ± 1.45 to 16.90 ± 1.65; r = 0.98, *p* < 0.001), reflecting expected weight restoration during Day Hospital treatment.

### 3.4. Associations Between Changes in Knowledge, Rules, and Eating Behavior

To examine whether cognitive–nutritional changes were associated with behavioral changes, correlations between pre–post change scores were computed. Changes in nutrient-related knowledge were positively associated with changes in fat exposure (Spearman’s ρ = 0.47, *p* = 0.01), whereas no significant association was observed with carbohydrate exposure. Changes in dietary rules were not significantly associated with changes in food exposure or habit-based measures.

### 3.5. Regression Analyses

Multiple linear regression models were conducted to examine the independent contribution of changes in nutritional knowledge and dietary rules to changes in eating behavior. The overall model explained 22.8% of the variance in changes in fat exposure (R^2^ = 0.228; adjusted R^2^ = 0.135) and approach, F(3, 41) = 2.86, *p* = 0.049. Changes in nutrient-related knowledge emerged as a significant independent predictor of changes in fat exposure (B = 0.69, SE = 0.26, *p* = 0.015; 95% CI [0.15, 1.23]), whereas changes in dietary rules and baseline BMI were not significant predictors. Multicollinearity diagnostics indicated no problematic collinearity (VIF range: 1.05–1.11). See Table 3 for details.

## 4. Discussion

This study examined pre–post changes in eating behavior, nutritional knowledge, and dietary rules during a structured, group-based nutritional psychoeducational program delivered within a Day Hospital setting for patients with anorexia nervosa. Participation in the program was associated with increased exposure to carbohydrate- and fat-containing foods, improvements in nutritional knowledge, reductions in rigid dietary rules, and weight gain during treatment. In addition, changes in nutrient-related knowledge were associated with changes in dietary fat exposure, independent of baseline BMI and changes in dietary rules.

Changes in eating behavior represent a clinically relevant outcome in anorexia nervosa, as restrictive dietary patterns often persist even during weight restoration [21,22]. The observed increase in the frequency of consumption of carbohydrate- and fat-containing foods is consistent with previous evidence indicating selective avoidance of specific macronutrients, particularly fats, in this population [16,22,23]. These findings support the importance of assessing food-specific eating behaviors, rather than relying solely on BMI as an indicator of nutritional change [10,24,25].

Nutritional knowledge showed substantial improvement over the observation period, particularly for nutrient-related content. Within cognitive–behavioral models of eating disorders, psychoeducation is conceptualized as a component supporting engagement with behavioral change, rather than as a therapeutic endpoint in itself [26,27,28]. In line with this view, the present study examined nutritional knowledge in relation to eating behavior rather than as an isolated outcome.

Dietary rules showed a marked reduction across domains, consistent with previous findings linking rigid rule-governed eating to illness severity and cognitive rigidity in anorexia nervosa [23,29,30]. However, changes in dietary rules were not directly associated with changes in eating behavior, suggesting that cognitive rule reduction and behavioral modification may occur concurrently but not necessarily in a tightly coupled manner during treatment. Changes in nutrient-related knowledge were associated with changes in dietary fat exposure in the regression model. However, given the modest sample size, the complete-case analysis, and the observational pre–post design without a control group, this finding should be considered hypothesis-generating rather than confirmatory.

The selective association between changes in nutrient-related knowledge and increased dietary fat exposure appears consistent with prior research indicating that fats are often perceived as particularly threatening and are disproportionately avoided in anorexia nervosa [22]. Although the observed pattern appears consistent with prior evidence indicating that fats are often perceived as particularly threatening and selectively avoided in anorexia nervosa, replication in larger and controlled samples is needed before drawing more definitive conclusions. Importantly, this association was not observed for carbohydrate exposure, suggesting that different food groups may be influenced by partially distinct cognitive and behavioral processes; however, this interpretation remains tentative. Accordingly, these findings should be interpreted cautiously and descriptively rather than causally.

### Limitations

This study has several limitations. First, the pre–post observational design without a control group precludes attribution of observed changes specifically to the nutritional psychoeducational program, as participants were concurrently receiving comprehensive multidisciplinary treatment. Therefore, the present findings cannot disentangle the contribution of the psychoeducational sessions from other therapeutic components of the Day Hospital program. Detailed data on psychiatric comorbidities, psychotropic medication use, and psychotherapy intensity were not systematically collected and could not be included in the analyses. These factors may have influenced behavioral and cognitive changes during Day Hospital treatment and should be considered in future research. Second, eating behavior was assessed using self-report measures, which may be subject to recall or reporting bias. Moreover, although the FFQ and the habit questionnaire served different purposes, both relied on self-report assessment, which may introduce shared-method variance and potential common-method bias. Third, the study-specific knowledge and habit questionnaire, although aligned with the intervention content and showing acceptable internal consistency for the total knowledge score, has not undergone independent psychometric validation and should therefore be considered exploratory. Fourth, the sample size limits statistical power for detecting smaller associations and does not allow for more complex modeling. Finally, only short-term changes were assessed; the persistence of changes after discharge remains unknown.

## 5. Conclusions

In a Day Hospital sample of patients with anorexia nervosa, participation in a group-based nutritional psychoeducational program co-occurred with specific changes in eating behavior, particularly increased exposure to carbohydrate- and fat-containing foods, alongside improvements in nutritional knowledge, reductions in dietary rules, and weight gain during treatment. Changes in nutrient-related knowledge were selectively associated with changes in dietary fat exposure. These findings underscore the potential value of the assessment of food-specific eating behaviors and cognitive–nutritional processes as complementary outcomes during treatment. Future controlled studies are needed to clarify the specific contribution of nutritional psychoeducation within multidisciplinary care.

## Figures and Tables

**Table 1:** Demographic and clinical characteristics at Day Hospital admission (N = 45).

Variable	Value
Age (years)	19.0 ± 5.2
Education (years)	12.4 ± 2.5
Duration of illness (months)	28.4 ± 28.2
BMI at admission	15.62 ± 1.21
Diagnosis, *n* (%)	
Anorexia nervosa, restricting type (AN-R)	42 (93.3%)
Anorexia nervosa, purging type (AN-P)	3 (6.7%)
Living situation, *n* (%)	
Living with family	35 (77.8%)
Other arrangements *	10 (22.2%)

Note. Values are reported as mean ± SD unless otherwise indicated. BMI at admission refers to BMI measured at entry into the Day Hospital program. Pre-intervention BMI (T0) is reported separately. * Includes living alone, with one parent, partner, or other family members. BMI = body mass index.

**Table 2:** Pre–post changes from the start (T0) to the end (T1) of the psychoeducational intervention.

Outcome	Pre (Mean ± SD)	Post (Mean ± SD)	Test Value	*p* (Holm)	Effect Size
Nutrient-related knowledge	11.49 ± 3.63	17.91 ± 3.15	*t* = 9.24 *	<0.001	dz = 1.38
Total nutritional knowledge	22.97 ± 5.98	32.24 ± 3.84	*t* = 9.35 *	<0.001	dz = 1.39
DRI total score	2.46 ± 0.73	1.85 ± 0.94	*Z* = −5.59 #	<0.001	*r* = 0.83
FFQ carbohydrates (times/week)	12.74 ± 7.91	16.62 ± 6.98	*t* = 4.61 *	0.003	dz = 0.69
FFQ fats (times/week)	8.74 ± 6.79	11.86 ± 5.58	*Z* = −3.04 #	0.014	*r* = 0.46
Carbohydrate exposure (habits score)	1.93 ± 0.75	2.33 ± 0.64	*Z* = −3.38 #	<0.001	*r* = 0.51
BMI	16.17 ± 1.45	16.90 ± 1.65	*Z* = −6.56 #	<0.001	*r* = 0.98

Note. Values are reported as mean ± SD. “Pre” refers to assessments conducted at T0 (one week prior to the start of the psychoeducational intervention), and “Post” refers to assessments conducted at T1 (one week after completion of the six-session program). BMI values in this table refer to pre-intervention BMI at T0 and post-intervention BMI at T1 and therefore differ from BMI at admission reported in Table 1. Holm’s correction was applied across primary outcomes. Effect sizes are reported as Cohen’s dz for paired *t* tests and r for non-parametric tests. * Paired-sample *t* test. # Wilcoxon signed-rank test. DRI = Dietary Rules Inventory; FFQ = Food Frequency Questionnaire; BMI = body mass index.

**Table 3:** Multiple linear regression model predicting changes in dietary fat exposure.

Predictor	β	SE	*p*	95% CI
Δ Nutrient-related knowledge	0.69	0.26	0.015	[0.15, 1.23]
Δ Dietary Rules Inventory	−1.07	1.42	0.456	[−3.99, 1.84]
Baseline BMI	−0.07	0.74	0.923	[−1.59, 1.45]

Note. Dependent variable: Δ FFQ fats (times/week). Positive β values indicate greater increases in weekly frequency of fat-containing foods. Δ indicates post–pre change scores.

## Data Availability

The datasets used and analyzed during the current study are available from the corresponding author upon reasonable request. The data are not publicly available due to privacy and ethical restrictions.
